# Selection of fast and slow growing bacteria from fecal microbiota using continuous culture with changing dilution rate

**DOI:** 10.1080/16512235.2018.1549922

**Published:** 2018-11-30

**Authors:** K. Adamberg, S. Adamberg

**Affiliations:** a Department of Chemistry and Biotechnology, Tallinn University of Technology, Tallinn, Estonia; b Center of Food and Fermentation Technologies, Tallinn, Estonia

**Keywords:** Dietary fiber, mucin, arabinogalactan, apple pectin, gut transit rate, colon

## Abstract

**Background**: Nutrient and energy metabolism in human colon depends on bacterial growth rate that is determined by the colonic transit rate.

**Objective**: A novel approach, De-stat culture was used to distinguish the fast and slow growing sub-populations from fecal microbiota.

**Design**: The enrichment and metabolism of bacteria from pooled fecal cultures of children was studied at dilution rates *D* = 0.2–0.0 1/h in mucin-supplemented media containing either arabinogalactan or apple pectin.

**Results**: The study revealed clear differentiation of the fecal microbiota at higher (above 0.1 1/h) and lower (below 0.1 1/h) dilution rates, along with metabolic changes. Similarity of the fast and slow growing bacteria was observed in two different fecal pools and on both substrates, suggesting the dilution rate as the main triggering parameter for selection of bacteria. At high dilution rates, the species *Collinsella aerofaciens, Dorea longicatena, Escherichia coli, Lachnoclostridium torques*, and different *Bacteroides* (*B. caccae, B. fragilis, B. ovatus, B. thetaiotaomicron, B. vulgatus*) were dominant in both media variants. At low dilution rates, *Akkermansia muciniphila, Eisenbergiella tayi, Negativicoccus succinivorans*, and a group of Ruminococcaceae became dominant in both media and in both fecal pools. This change in bacterial population accompanied by the increased production of propionic and butyric acids as well as higher consumption of alanine and branched chain amino acids at low dilution rates.

**Conclusions**: The study suggests that specific growth rate has important effect on the dynamics of colon microbiota. Manipulation of the proportions of fast and slow growing gut bacteria through modulation of the transit rate could be a target in human nutrition studies. The De-stat study would enable to predict changes in microbiota composition associated with the decrease or increase of the colonic transit rate.

## Introduction

The gastrointestinal microbiota has an important role in human health with impact on nutrition, immune functions, and physiology [1]. The metabolic activity of the gut microbiota is equally important as its diversity, the suggested indicator of gut health [2]. Dietary fibers serve as substrates for colon bacteria but also influence the important physiological processes of the bowel such as peristalsis and transit rate. The fibers that are only partially or non-fermented by the gut microbiota increase the fecal volume and transit rate by binding to water. Peristalsis, water absorption, environmental acidity, redox potential, and the absorption of nutrients are affected by the intestinal microbiota, and in turn, affect the microbiota as well [3,4]. Hence, short bowel transit time (below 24 h) might support the fast-growing bacteria and can be a key factor to control the fermentation of dietary fibers in the colon. The study of Roager revealed associations of the colonic transit time and the overall gut microbial composition, diversity, and metabolism [4]. The longer colonic transit time was found to accompany with the higher microbial richness and a shift in colonic metabolism from carbohydrate fermentation to protein catabolism, while shorter transit time correlated with the metabolites possibly reﬂecting the increased renewal of the colonic mucosa. For instance, increased butyrate concentrations in distal colon were detected at faster transit [5].

Various microbial shifts have been observed to accompany with the long retention time. Zhu et al. reported association between long retention time and decrease of relative abundance of *Prevotella* [6]. In two other studies, the increase of the transit time resulted in the decreased amounts of *Bifidobacterium* and *Bacteroides* [7] or *Roseburia, Pseudobutyrivibrio,* and *Megamonas* [8].

Various *in vitro* models of the gut microbiota have been used to examine the effects of prebiotics [9,10], probiotics [11], and dietary modulations [12] on the gut microbiota and its metabolites. *In vitro* models facilitate frequent sampling and increased reproducibility [13]. Despite clear associations between the colonic transit time and microbiota, the effect of specific growth rate on the composition and metabolism of microbiota is not studied thoroughly *in vitro*. More extensive use of chemostat or fed-batch cultures in bowel ecological research would help to generate answers to very fundamental questions as to how the bowel microbial community is regulated by substrate affinities and energetics, and how the community will respond to altered dietary composition [14]. Moreover, although specific substrate consumption ability is required for the growth of given bacteria, the concentration of the substrate in the environment is also important. In natural ecosystems the substrate concentrations are usually too low to allow maximal growth rate to be achieved and batch cultures do not predict ecological outcome in bacterial communities [15]. Therefore, dynamic data are required to predict bacterial behavior in communities such as colon microbiota. Change-stat method is a modification of the classical chemostat or turbidostat cultures. The unifying concept behind different change-stats is the smooth continuous adjustment of an environmental parameter within a single experiment, while maintaining the physiological state of the culture practically stable [16].

Modification of the dilution rate *in vitro* has been shown to affect the establishment of certain types of bacterial communities together with the changes in their metabolic activities [17]. For instance, dilution rate 0.08 1/h increased the proportion of lactobacilli and bifidobacteria compared to that at 0.04 1/h [18] and in another study more organic acids were produced at *D* = 0.1 1/h than at *D* = 0.04 1/h [19]. In addition, very low dilution rates (0.02 1/h) were shown to decrease the biomass concentration and bacterial diversity in a simulated colonic model [20].

In the gut, significant amount of energetic amino acids and polysaccharides is obtained from mucins – heavily glycosylated proteins secreted by gut epithelial cells. The secretion rate, composition, and thickness of the mucin layer vary along the gastrointestinal tract and are controlled by several factors, including the feed-back (metabolites) from the gut microbiota [21,22]. These aspects cannot be adequately reproduced in most laboratory gut models. Although the epithelium is not in contact with the gut lumen, the mucus is continuously produced and can be degraded by mucosal bacteria such as *Akkermansia* and others, providing oligosaccharides and peptides also for luminal bacteria [21]. Thus, we supplemented the growth medium with mucin to cover partly its effect on the bacterial dynamics and metabolism.

The objective of the current study was to evaluate the applicability of a continuous cultivation with gradual decrease of the dilution rate (De-stat) to select preferentially fast or slow growing sub-populations from the fecal microbial consortia. Apple pectin (AP) and arabinogalactan (AG) as common food fibers from apples, plums, and cereals (rye, wheat) in Estonian diet were used as selective substrates and two fecal pools of children were used as inocula to test the method.

## Materials and methods

### Fecal inocula

The fecal samples were collected from normal weight (NW, age 9.9 ± 4.3 years), and overweight (OW, age 9.8 ± 2.7 years, body mass index over the 95 percentile indicating that all children were obese) children. The fecal samples were diluted with protectant-containing solution 1:4 as described in Adamberg et al. [23] and kept at −80°C until use. Before cultivation experiment, the fecal slurries were thawed, and pooled in equal volumes as also reported earlier by Aguirre et al. [24] The OW pool for inoculation consisted of seven fecal slurries of the OW children (4 boys, 3 girls) and the NW pool consisted of six fecal slurries of the NW children (2 boys, 4 girls). The average age and range of the fecal donors were 10.6 ± 2.4 (range 7–14 years) and 9.4 ± 4.7 (range 4.5–15 years) for OW and NW fecal pools, respectively.

### Defined base medium

The defined growth medium contained components as follows: 0.05 M potassium phosphate buffer made from 1 M stock solutions (mL/L): K_2_HPO_4_ (28.9) and KH_2_PO_4_ (21.1); mineral salts (mg/L): MgSO_4_·7H_2_O (36), FeSO_4_·7H_2_O (0.1), CaCl_2_ (9), MnSO_4_·H_2_O (3), ZnSO_4_·7H_2_O (1), CoSO_4_·7H_2_O (1), CuSO_4_·5H_2_O (1), MgCl_2_ (2), (NH_4_)_6_Mo_7_O_24_·4H_2_O (1), NaCl (527), (NH_4_)Cl (400); and 10 mL/L hemin – vitamin K_1_ solution (hemin – 5 mg/L and vitamin K_1_ 0.5 mg/L in final medium) and amino acids (g/L): Ala (0.044), Arg (0.023), Asn (0.038), Asp (0.038), Glu (0.036), Gln (0.018), Gly (0.032), His (0.027), Ile (0.060), Leu (0.120), Lys-HCl (0.080), Met (0.023), Phe (0.050), Pro (0.041), Ser (0.095), l-Thr (0.041), Trp (0.009), Val (0.060), Tyr (0.015); pH 7.2 ± 0.1. The carbohydrate substrates were sterilized separately and mixed with the medium before cultivation. Two substrate combinations, either AG (Sigma-Aldrich, USA) or AP (Sigma-Aldrich, USA) with porcine mucin (Type II, Sigma-Aldrich, USA) were added to the base medium in equal amounts (2.5 g/L each).

### Cultivation method

The Biobundle cultivation system consisted of ADI 1030 bio-controller and cultivation control program ‘BioXpert’ (Applikon, Delft, The Netherlands). The fermenter was equipped with sensors for pH, pO_2_, and temperature. The variable speed pumps for feeding and outflow were controlled by De-stat algorithm, *D* = *D*_0_ − *d* × *t*, where *D* is the dilution rate (1/h), *D*_0_ is the initial dilution rate, *d* is the deceleration rate (1/h^2^), and *t* is the time (h). pH was controlled by acid and base addition according to pH set-point. The culture volume was kept constant (300 mL) by monitoring the weight of the fermenter with the PC-linked balance and excess of liquid was removed by outflow pump. The pH of the culture was kept at 7 and temperature at 36.6°C. The medium was pumped in the fermenter and the medium bottle and fermenter were flushed with the nitrogen gas overnight to remove all oxygen from the system. The nitrogen flushing was on during the whole experiment. Next morning, 2 mL of the pooled fecal culture was inoculated.

**Figure 1.**
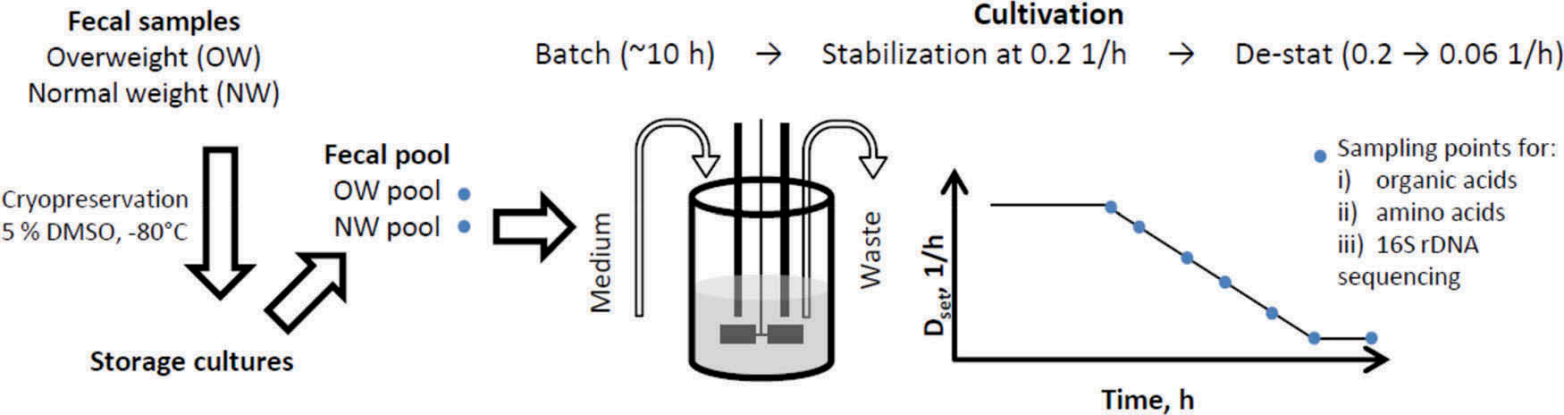
Experimental scheme of the De-stat cultivations. The pooled fecal inoculum was added into fermenter at time 0 h followed by batch phase (ca 10 h) until beginning of exponential growth. Then continuous mode was started and after the stabilization of the fecal culture at *D* = 0.2 1/h (6–7 residential times), the dilution rate was gradually decreased down to 0.06 1/h (deceleration rate 0.05 1/h per day) followed by re-stabilization for approximately 2 residential times. The same procedure was applied for two substrate combinations (arabinogalactan + mucin, apple pectin + mucin) and for two inocula: the pooled fecal samples of the overweight and normal weight children. *D*_set_ indicates the dynamics of the pre-set dilution rate.

Eight-to-ten hours after inoculation (exponential growth of bacteria was detected by decrease of pH), the cultivation algorithm was started and dilution rate was increased smoothly to 0.2 1/h for stabilization of the culture at this value by 5.5–7 residence times. After stabilization (stable gas production and titration rate), the dilution rate was decreased down to 0.06 1/h at a rate of 0.05 (dilution rate) units per day (experimental timeline is presented in the Figure 1). The dilution rate interval was chosen to support the realistic growth rates of luminal bacteria in the colon. Specific growth rate of the bacteria was calculated based on the colonic transit time of digesta in people consuming the Western diet, which varies from 40 to 140 h (median 60–70 h) [25–27], and the estimated amount of bacteria, that increases from 10^8^ in proximal colon to 10^11^ cfu/g in feces [28]. Considering both the period the bacteria have for degradation of the dietary fibers and the coinciding increase of the bacterial biomass in colon, the specific growth rate of the bacteria decrease from 0.3 down to 0.02 1/h.

### Analytical methods

Samples from the outflow were collected on ice, centrifuged (14,000 g, 5 min, 4°C), and stored separately as pellets and supernatants at −20°C until HPLC analyses (sugars and organic acids), UPLC analyses (amino acids), and microbial 16S rDNA sequencing.

For chromatographic analysis, the supernatants were ultra-filtered using AmiconR Ultra-10K Centrifugal Filter Devices, cut-off 3 kDa (Millipore, Cork, Ireland). The concentrations of organic acids (succinate, lactate, formate, acetate, propionate, isobutyrate, butyrate, isovalerate, valerate), glycerol, ethanol, and free sugars (mono-, di- and trisaccharides) were determined by high-performance liquid chromatography (HPLC, Alliance 2795 System, Waters, Milford, MA, USA), using a BioRad HPX-87H column (Hercules, CA, USA) with isocratic elution of 0.005 M H_2_SO_4_ at a flow rate of 0.5 mL/min and at 35°C. Refractive index (model 2414; Waters, USA) and UV (210 nm; model 2487; Waters, USA) detectors were used for quantification of the substances. Detection limit for the HPLC method was 0.1 mM.

Concentrations of amino acids and amines were determined with an amino acid analyzer (UPLC; Waters, Milford, USA) according to the manufacturer’s instructions. Detection limit of the method was 0.01 mM. All standard substrates were of analytical grade. Empower software (Waters, USA) was used for the processing of HPLC and UPLC data.

Composition of the gas outflow (H_2_, CO_2_, H_2_S, CH_4_, and N_2_) was analyzed using the Agilent 490 Micro GC Biogas Analyzer (Agilent 269 Technologies Ltd., Santa Clara, CA, USA) connected to the thermal conductivity detector. The volume of the gas flow was recorded by MilliGascounter (Ritter, Bochum, Germany).

Redox potential of the growth medium and culture supernatant was measured by pH/Redox meter using InLab®Redox electrode (Mettler Toledo, Schwerzenbach, Switzerland).

Biomass dry weight was measured gravimetrically by centrifuging the biomass from a 10 mL culture, washing quickly twice with distilled water and drying at 105°C for 24 h in an oven.

### DNA extraction and amplification

DNA was extracted from the pellets using PureLink Microbiome DNA extraction kit (Thermo Fisher Scientific, Carlsbad, CA, USA) according to the manufacturer’s instructions. Universal primers:

S-D-Bact-0341-b-S-17 Forward (5´ TCGTCGGCAGCGTCAGATGTGTATAAGAGACAGCCTACGGGNGGCWGCAG) and S-D-Bact-0785-a-A-21 Reverse (5´ GTCTCGTGGGCTCGGAGATGTGTATAAGAGACAGGACTACHVGGGTATCTAATCC) were used for PCR amplification of the V3–V4 hypervariable regions of the 16S rRNA genes [29]. The amplified region was 390–410 bp long and average 67,000 reads per sample were obtained. The mixture of amplicons was sequenced using Illumina MiSeq 2× 250 v2 platform (Estonian Genome Centre, University of Tartu, Estonia).

### Taxonomic profiling of fecal samples

DNA sequence data was analyzed using BION-meta (www.box.com/bion), currently unpublished open source program, according to author´s instructions. First, sequences were cleaned at both ends using a 99.5% minimum quality threshold for at least 18 of 20 bases for 5′-end and 28 of 30 bases for 3′-end, then joined, followed by removal of shorter contigs than 350 bp. Then, sequences were cleaned from chimeras and clustered by 95% oligonucleotide similarity (k-mer length of 8 bp, step size 2 bp). Lastly, consensus reads were aligned to the SILVA reference 16S rDNA database (v123) using word length of 8 and similarity cut-off of 90%. The bacterial designation was analyzed at different taxonomic levels down to species if applicable.

### Calculations

For quantitative data analysis, first the relative data of bacterial abundances from 16S rDNA sequencing analysis were converted to quantitative values (*X_i_* [g/L] where *i* illustrates bacterial taxa *i*) by formula: *X_i_* = *X_t_* × *A_i_*, where *X_t_* is the dry weight of total biomass of bacteria (g/L) and *A_i_* is the relative abundance of bacterial taxa *i* in the sample.

The growth characteristics of the bacteria in A-stat experiments were calculated on the basis of bacterial mass, total volume of medium pumped out from fermenter (*V*_OUT_, L) and product concentrations in culture medium (mol L^−1^) as follows:

(1) $$\mu =\frac{d(V_{OUT})}{V\times dt}+\frac{d(X_{t})}{dt\times X_{t}}\underset{x\to \infty }{\lim}$$

(2) $$Q_{S}=\frac{S\times d(V_{OUT})}{V\times Xt\times dt}-\frac{d(S)}{dt\times Xt}$$

(3) $$Q_{Pi}=\frac{Pi\times d(V_{OUT})}{V\times Xt\times dt}+\frac{d(Pi)}{dt\times Xt}$$

where *μ* is the specific growth rate (h^−1^); *Q_S_* is the carbohydrate consumption rate (in carbon equivalents, mol-C/g-*X_t_*/h); *S* is the concentration of consumed carbohydrates (C-mol/L); *Q_Pi_* is the production rate of product *i* (mol-prod g-X^−1^ h^−1^); *V* is the current fermenter volume (L); *V*_OUT_ is the outflow volume, and *t* is the cultivation time (h).

For diversity analysis, Shannon index (*S_i_*) was calculated using formula: *S_i_ *= ∑*p_i_* × ln(*p_i_*), where *p_i_* is the relative abundance of bacterial taxa *i* in the sample.

### Statistical analyses

To compare differences in bacterial abundances and metabolite productions during De-stat experiments samples were divided into two groups: (i) samples taken at *D* < 0.1 1/h (slow growth) and (ii) samples taken at *D* > 0.1 1/h (fast growth). Average and standard deviation of bacterial abundances and metabolite productions were calculated in both groups and single parametric *t*-test was used to estimate statistical significance.

### Ethics statement

The study was approved by the Tallinn Medical Research Ethics Committee, Estonia (protocol No. 554) and by the Research Ethics Committee of the University of Tartu, Estonia (protocol No. 215/T-21).

## Results

### Dilution rate dependent bacterial dynamics of fecal microbial cultures

Compared to the OW fecal pool, the NW fecal pool had higher relative abundance of *Akkermansia muciniphila* (4.1 ± 0.8% vs 0.9 ± 0.6%, respectively, *p*-value 0.05) while lower abundances of *Eubacterium rectale* (1.8 ± 0.0% vs 2.9 ± 0.2%, respectively, *p*-value 0.01) and *Bifidobacterium adolescentis* (6.5 ± 0.3% vs 10.4 ± 2.0%, respectively, *p*-value 0.11, Table 1). Above analytical threshold of 0.01%, 149 vs 130 bacterial species were detected in the fecal pool of the NW and OW children, respectively (Table 1 and Supplementary Table S1). The microbial diversity expressed as Shannon index was also higher in the NW compared to that of the OW fecal pool (3.80 ± 0.09 vs 3.63 ± 0.02, respectively).

**Table 1.** Ten most abundant species (shown in bold) and the species significantly different between the fecal pools of the normal weight (NW) and overweight (OW) children (abundance ± standard deviation and *p*-value of single parametric *t*-test). All samples were analyzed in two parallels. Additional data can be found in Supplementary Table S1.

Species	OW	NW	*p*-Value
*Faecalibacterium prausnitzii*	8.8 ± 0.4	8.3 ± 0.1	0.25
*Bifidobacterium adolescentis*	10.4 ± 2	6.5 ± 0.3	0.11
*Holdemanella unclassified*	8.2 ± 0.8	8.6 ± 3.3	0.87
*Catenibacterium mitsuokai*	6.3 ± 1.5	5.6 ± 0.3	0.58
*Bifidobacterium pseudocatenulatum*	3.5 ± 1	3 ± 0.4	0.54
*Collinsella aerofaciens*	3.2 ± 0.5	3.3 ± 0.1	0.81
*Bifidobacterium longum*	2.9 ± 0.2	3 ± 0.1	0.69
*Dorea longicatena*	3 ± 0	2.5 ± 0.2	0.08
*Akkermansia muciniphila*	0.9 ± 0.6	4.1 ± 0.8	0.05
*Eubacterium rectale*	2.9 ± 0.2	1.8 ± 0	0.01
*Anaerostipes hadrus*	0.6 ± 0	1.5 ± 0.1	0.00
*Barnesiella intestinihominis*	0.03 ± 0.01	0.25 ± 0.01	0.002
*Bacteroides thetaiotaomicron*	0.02 ± 0.01	0.23 ± 0.01	0.002
*Bacteroides ovatus*	0 ± 0	0.48 ± 0.07	0.011
*Bacteroides uniformis*	0.06 ± 0.04	0.59 ± 0.11	0.022
*Lachnoclostridium lactaris*	0.11 ± 0	0.49 ± 0.09	0.026
*Bacteroides vulgatus*	0.19 ± 0.14	0.71 ± 0.06	0.041
*Senegalimassilia anaerobia*	0.16 ± 0.07	0.43 ± 0.05	0.047

**Figure 2.**
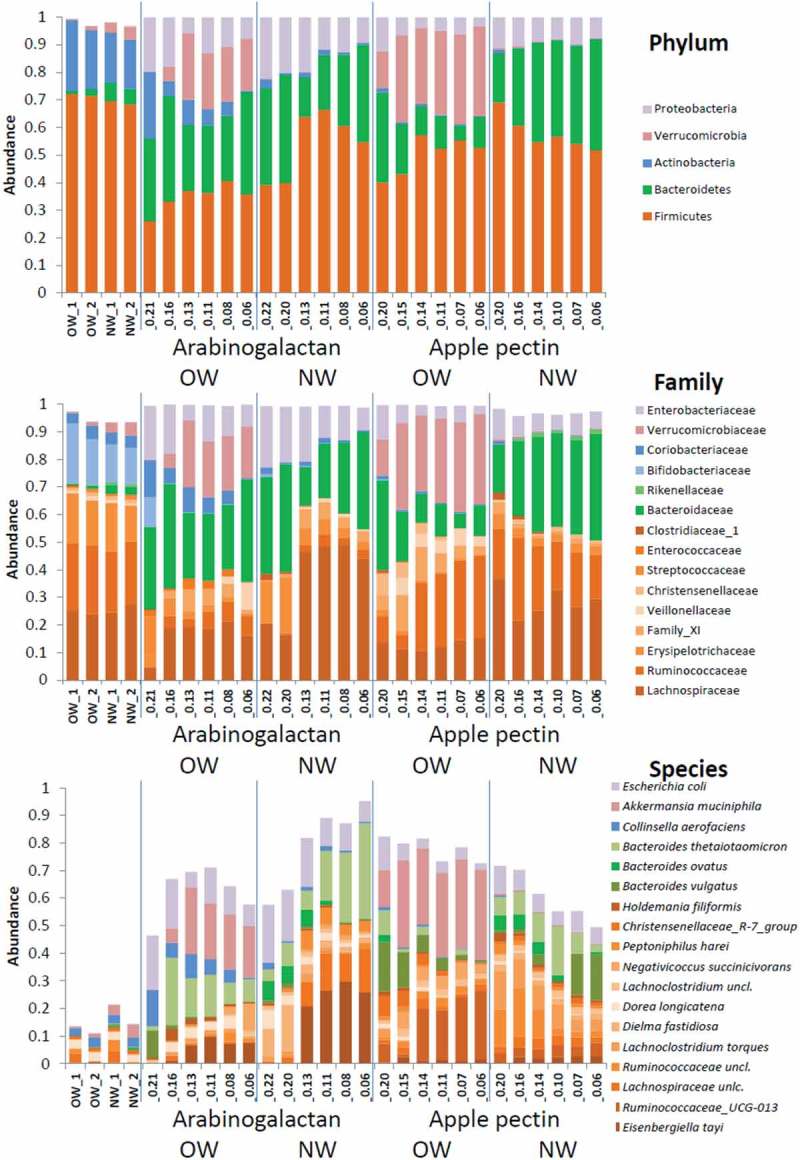
Composition of fecal microbiota in pooled inocula of the overweight (OW) and normal weight (NW) children, and during the De-stat cultivation on arabinogalactan and apple pectin in mucin-supplemented medium on three taxonomic levels. Values at the *x*-axes designate the dilution rates of the sampling points. Only taxa having average abundance more than 1% are shown in the graphs.

In Figure 2, the microbial dynamics within the range of the studied dilution rates is presented on three taxonomic levels, phylum, family, and species, while differences can be seen mainly below the family level as also shown by Chung et al. [9] The main phylum in both inocula, Firmicutes, remained dominant throughout the cultivation experiments. In De-stat cultures, the proportion of Bacteroidetes was higher at low dilution rates (*D* < 0.1 1/h) but not significantly in the NW culture on AP, while decrease was observed in the OW cultures on this substrate. The amount of generally fast-growing Proteobacteria and Actinobacteria mostly on AG was higher at high dilution rates in all experiments. Rise in proportions of Verrucomicrobia was clearly seen in the OW cultures on both substrates. On the family level, Ruminococcaceae and Lachnospiraceae (Firmicutes) composing half of the initial populations remained dominant also during the whole cultivations; however, Ruminococcaceae increased with decrease of dilution rate significantly (*p* < 0.05). Bacteroidaceae containing several species was the only family of Bacteroidetes detected in De-stat cultures.

The biggest changes were observed on the species level, 53–78% from the total reads belonged to species that formed less than 10% of the initial population, while some of the minor species (incl. several *Bacteroides* species) became prevalent at *D* = 0.2 1/h (Figure 2, Table 1). Generally, AP supported higher diversity of fecal microbiota while AG exhibited somewhat higher selectivity (lower diversity and Shannon index) under decreasing dilution rate conditions (Supplementary Table S1). About two times decrease of the initial richness of the species (abundance above 0.01%) was seen in both media (containing either AG or AP) from 118 and 133 species down to 60 and 70 species, in OW and NW cultures, respectively. The fast-growing species in all cultures were *Escherichia coli* and several species of *Bacteroides*, *Dorea*, and *Lachnoclostridium* (Figure 2). Some species were characteristic mostly to the OW (*Collinsella aerofaciens, Lachnoclostridium lactaris*) or to the NW cultures (*Bacteroides ovatus, Peptoniphilus harei*) but no clear substrate specificity (AP or AG) was observed. In our study, the amount of *A. muciniphila* was higher in the pooled fecal microbiota of the NW children. However, during continuous cultivation in media containing either AG or AP, the amount of *Akkermansia* increased only in the OW fecal cultures.

### Metabolism of arabinogalactan and apple pectin in the presence of mucin

#### Production of organic acids and gases

**Figure 3.**
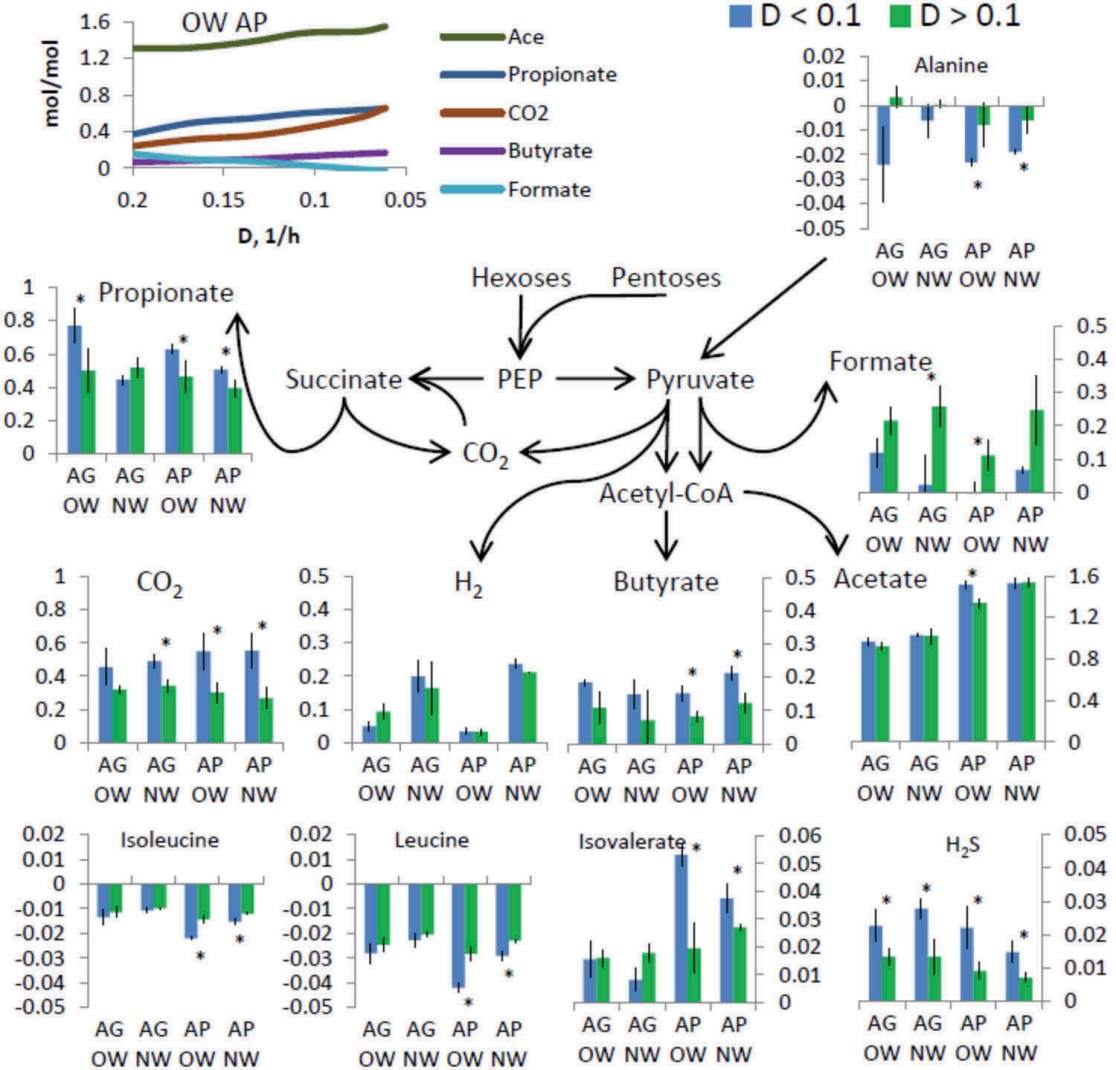
Production of organic acids and gases and consumption of amino acids per carbohydrates consumed (molar yields, all sub-figures have *x*-axis unit mol/mol) in De-stat cultures with pooled fecal inocula of children. On the top left figure, the metabolic products during the decrease of dilution rate from 0.2 to 0.06 1/h, are shown in experiment of OW fecal pool and AP mucin medium as an example. In the other plots average values of productions or consumptions at high (>0.1 1/h) or low (<0.1 1/h) dilution rates are shown for all experiments. Metabolic scheme illustrates overall picture without cross-feeding possibilities between different bacteria. OW – pooled fecal samples of the overweight children, NW – pooled fecal samples of the normal weight children, AG – arabinogalactan, AP – apple pectin, ace – acetic acid, but – butyric acid, for – formic acid, ibut – isobutyric acid, lact – lactic acid, prop – propionic acid, succ – succinic acid, val – valeric acid. Mucin was present in all growth media. The blue bars depict the interval of the dilution rate from 0.06 to 0.1 1/h, and the green bars – dilution rate *D* from 0 0.1 to 0.2 1/h. Asterisks indicate *p*-value on single parametric *t*-test of molar yields between high and low dilution rates. Production and consumption rates of all metabolites (mmol/g/L) are given in Supplementary Table S2.

The metabolite patterns were related to the substrates and dilution rates, as well as respective changes in microbiota composition. Based on HPLC analysis, no free sugars were detected in the culture liquid and at least 90% of acids from carbohydrates present in the medium were formed in cultivation. Acetic acid, the most abundant organic acid in the fecal cultures (43–67% of total acids), was produced in amounts 0.75–1.43 mol per one mole of carbohydrate consumed (Figure 3, Table S2). Production of acetate remained stable throughout the decrease of the dilution rate, yet, being about 1.5 times higher in case of AP compared to AG. On the contrary, production of less abundant acids such as butyrate, formate, and propionate was clearly decreased at lower specific growth rates and fecal pool-specific responses were seen. The main gases detected were hydrogen and carbon dioxide, which accompanied with the production of propionic and butyric acids (Figure 3). Less propionic acid (mol/mol carbohydrate) and more hydrogen was produced from AG in the NW pool than in the OW pool. Along with the decrease of the dilution rate from 0.2 to 0.06 1/h, the production of propionic acid was increased up to more than twice (from 0.36 up to 0.87 mol/mol AG) in the OW fecal culture. In these cases, higher concentrations of propionate were in accordance with the increased numbers of propionate-producing bacteria such as *Akkermansia, Negativicoccus*, and *Bacteroides*. For example, in the OW cultures stabilized at *μ* = 0.06 1/h, *B. caccae, B. thetaiotaomicron*, and *B. xylanisolvens* constituted up to 35% from the detected taxa (Tables S1 and S2).

The yield of butyrate remained below 0.1 mol/mol carbohydrate metabolized at dilution rate *μ* = 0.2 1/h, but increased to about 0.2 mol/mol at *μ* = 0.06 1/h in all cultures along with notable enrichment of the butyrate producing species *Eisenbiergiella tayi* and *Faecalibacterium prausnitzii* (Table S1). Additionally, other slow growing but less abundant butyrate producing species *Coprococcus comes, Flavonifractor plautii*, and *Eubacterium oxidoreducens* were detected in these cultures. Higher propionate and butyrate production was accompanied by reduced biomass yields in AP medium 24% and 35% in NW and OW cultures, respectively, but not in AG medium (Table S2).

Production of formic acid was characteristic to high specific growth rates yielding 0.2 mol/mol in the OW and 0.3 mol/mol in the NW fecal pools. The gas data suggest possible formate conversion to carbon dioxide and hydrogen at low dilution rates (Figure 3, Table S2) that was accompanied by reduced amounts of *Lachnoclostrium* species, known to be involved in formate production. Another fermentation product associated with higher growth rates (>0.1 1/h) was succinic acid. Elevated succinate concentrations were detected in the OW fecal cultures, however, not significantly different those in the NW fecal pools. This could refer to a limited conversion of succinate to propionate in the OW pool growing at high dilution rates. Indeed, in the OW cultures growing at low growth rates in the AP-containing medium, the abundance of succinate-metabolizing bacteria such as *Negativicoccus* increased up to 5% from total reads.

#### Amino acid metabolism

Free amino acids providing 20% of total carbon in the medium were completely consumed (not detected in the spent medium, Table S2) by both fecal pools and on both substrate combinations, except for alanine, phenylalanine, and branched chain amino acids (BCAA) within the whole range of dilution rates studied. Accordingly, isovalerate, a degradation product of leucine was detected at low dilution rates in the medium containing AP. In the same medium, more intensive metabolism of alanine occurred at low dilution rates with maximum requirement of 23 ± 1.5 mmol per mol of carbohydrates (mmol-AA/mol-carb) in the OW fecal culture and 18.8 ± 1.2 in the NW fecal culture, respectively (Figure 3). An up-shift in alanine consumption was observed below specific growth rate 0.13 1/h and was more pronounced in the AG containing medium. Consumption of alanine was higher in the OW compared to that in the NW cultures. In addition, consumption of isoleucine, leucine, valine, and phenylalanine increased at low dilution rates.

## Discussion

### Change-stat as a novel approach in microbiota research

Batch culture is the simplest method to study the fermentation of (dietary) polysaccharides, with clear advantages of small volume and low cost, and thus, suitability for screening experiments. However, the physiological state of complex microbial consortia in dynamic environments can be simulated only in computer-controlled continuous cultures. Fed-batch with feeding to decrease specific growth rate might be more relevant in simulating conditions in colon (increase of biomass concentration and decrease of specific growth rate from proximal toward distal colon); however, to obtain the response from single parameter such as specific growth rate we propose the De-stat approach with decreasing dilution rate from 0.2 down to 0.06 1/h to study the microbial dynamics in the continuously changing conditions of specific growth rate. By using a classical chemostat, the effect of a single parameter at a constant dilution rate can be studied at a time, while a run of the De-stat makes it possible to study the wide range of dilution rates and select several bacterial sub-populations in addition to substrate selectivity also by different growth rate preferences. Thus, it is time and resource saving to run a single De-stat instead of several chemostat experiments. It should be emphasized that the main aim of using change-stat is not to obtain fully stabilized abundances of bacteria at a certain dilution rate, but rather to gain information about adaptive response of bacterial consortia to the changing dilution rate. Different change-stat and accelerostat techniques have been applied in pure culture studies earlier [16]. The De-stat culture has not been applied for studies of complex microbial consortia earlier, while chemostat gut models have been used by several groups. It has been shown that major shifts in variability of fecal microbiota take place within the first 3–4 residential times while slight fluctuations occur even after 30 residential times [13,20,30] because of complex nature of the microbiota. Hence, the De-stat presented in the current paper is a promising approach for analyzing of the growth rate in microbial consortia with potential impact on health.

To inoculate the repeated/parallel fermentation experiments, we used the frozen (−80°C) stock suspensions/cultures of fecal microbiota containing dimethylsulfoxide as protectant, also suggested by Gaci et al. [31] Functional preservation of the fecal samples has several advantages over using fresh fecal material, as composition of the colon/fecal microbiota varies from day to day. Therefore, use of the same inoculum would afford better reproducibility of the fermentation experiments. In different from Gaci et al. [31], we preserved the diluted feces with added protective agent without any batch cultivations to save the original microbial balance and functionality.

### Dilution rate dependent differentiation of the fast and slow growing sub-populations

**Figure 4.**
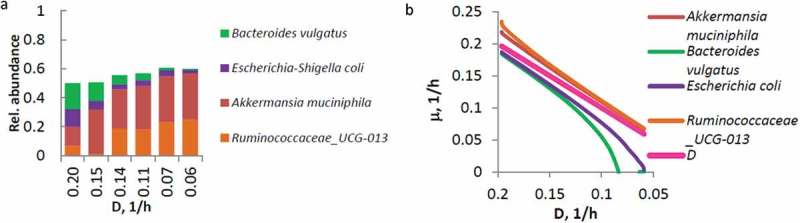
The dynamics of fecal microbiota in De-stat cultures. In (A), an example of the distribution of relative abundances of the selected species under dilution rates *D* = 0.2–0.06 1/h, with the data of the fecal pool of the overweight children growing in medium containing apple pectin (AP) and mucin. (B) Responses of the specific growth rates of the species, presented in (A), to the changing dilution rate. The species classified as only fast-growing bacteria, did not follow the pre-set decrease of the dilution rate (below the D line) and were washed out from the fermenter.

**Figure 5.**
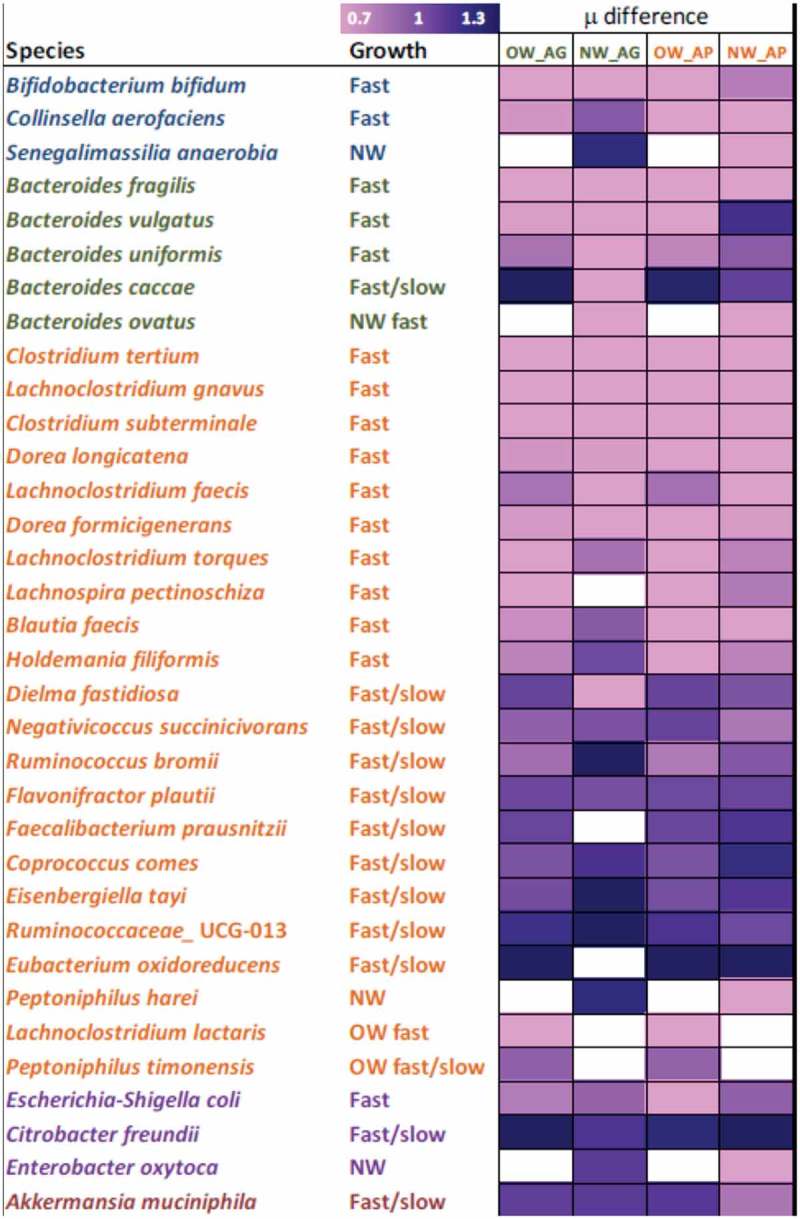
Based on the differences in specific growth rates shown in Figure 4, the species are divided in two groups: (1) the species growing only at high dilution rates and washed out at low dilution rate (indicated in pink) and (2) the species able to follow the pre-set dilution rate (indicated in dark blue). The color intensity indicates how many times in average the specific growth rate of a species differs from the pre-set dilution rate. OW and NW indicate the fecal pools from the overweight or normal weight children; AG and AP – arabinogalactan and apple pectin, respectively. Color code of the species list designates the phyla: blue – Actinobacteria, green – Bacteroidetes, orange – Firmicutes, violet – Proteobacteria, brown – Verrucomicrobia. The species are sorted based on phylum and then based on relative difference of specific growth rate and pre-set dilution rate (only the fast-growing species first). Only statistically different changes are shown. Specific growth rates are calculated from the amount of a species detected under corresponding dilution rate using formula (1)–(3).

Based on the estimated differences between the pre-set dilution rate and specific growth rate, the bacterial species were divided in two groups: the bacteria able to grow at high dilution rates only (washed out at lower dilution rates, i.e. end of the De-stat) and the bacteria growing equally well at high and slow growth rates (the species that followed or exceeded the pre-set dilution rate) (Figures 4 and 5). For example, with the decrease of dilution rate, decrease of *B. vulgatus* and *E. coli*, while increase of *A. muciniphila* and *Ruminococcaceae* were seen in the OW cultures in AP-containing medium (Figure 4). In parallel with the decreasing dilution rate, the diversity of the microbiota declined as the species selected only at high dilution rates started to be washed out (Figure 5). It should be considered that, depending on the growth rate, different species may possess different substrate affinities, and the species with the highest affinity for the substrate will outcompete other species at low substrate concentrations [15]. In addition, when several substrates are co-fermented, possibly cross-feeding and communication would occur. The number of the fast-growing species (abundance over 0.1%) remained between 19 and 24 at lower dilution rates (*μ* < 0.1 1/h), depending on the substrate and type of inoculum.

One of the crucial factors influencing the specific growth rate of bacteria in the colon is the transit rate of undigested food residues. The modulating effect of specific growth rate on the colon microbiota is not understood, yet, however, it can be assumed that faster transit supports fast-growing bacteria and *vice versa*. Roager et al. showed that long transit time is associated with higher abundances of *Methanobrevibacterium* and certain taxa from *Ruminococcaceae* and *Christensenellaceae* in adult fecal microbiota [4]. Despite the significance of the transit time, this parameter is difficult to measure in practice. This is usually estimated indirectly by a Bristol stool scale (BSS). Indeed, in the large population-based cohort (*n* = 1126, average age 45 years, LifeLines-DEEP project), association between harder stools (characteristic to long transit time) and *Ruminococcaceae, Christensenellaceae*, and several *Proteobacteria* [32] were observed. The same taxa and *Akkermansia* were related to harder stools in another study (*n* = 56) [33].

The species growing within the whole range of dilution rates and, on both sets of substrates, comprised *Coprococcus comes, Faecalibacterium prausnitzii, Flavonifractor plautii*, and a group of Ruminococcaceae UCG-013 (Figure 5). Most interestingly, some species from the genera *Eisenbergiella, Eubacterium oxidoreducens* group, and *Negativicoccus*, that were not detected in the pooled fecal inocula (detection limit of 0.01%), became dominant at low dilution rates. For example, at *D* = 0.06 1/h, *Eisenbergiella tayi* composed 8% and 26% in AG and mucin medium in the OW and NW fecal pools, respectively.

At low dilution rates, also *Eubacterium coprostanoligenes, Eisenbergiella tayi*, and *Negativicoccus succinivorans* were selected (Figure 5). *Eisenbergiella* is a lately described genus isolated from a sepsis sample [34,35]; however, occurrence of similar bacteria have been reported in stool samples [34,36]. According to our knowledge, data about cultivation of *Eisenbergiella* species in different environmental conditions are scarce. Amir et al. [34] showed that *Eisenbergiella tayi* was asaccharolytic and produced acids (about half was butyrate and the rest lactate, acetate, and succinate) only from glucose in tryptone peptone medium. However, this species showed positive reactions for several glycoside hydrolases showing potential for carbohydrate degradation. *Negativicoccus succinicivorans* is a novel little studied bacterium; however, succinate has been shown to support the growth of this bacterium [37]. Succinate can be produced by several gut bacteria such as *Bacteroides* species. Thus, high abundance of *Bacteroides* species (10–50%) in our experiments may explain the promoted growth of *Negativicoccus* at low dilution rates.

### Slow growth favors the production of butyric and propionic acids along with increased consumption of amino acids

The bacterial dynamics was reflected in metabolite patterns as butyrate and propionate-producing bacteria preferably grew at low dilution rates. Enhanced propionate production at low specific growth rates was also reported by us and others in pure cultures of *B. thetaiotaomicron* [38,39,40]. Production of propionate enables to save carbon and regenerate NAD^+^ through reductive TCA cycle which can be coupled with anaerobic respiration, that is more efficient compared to acetate production through phosphoketolase reaction, with the respective ATP yields 4 mol/mol glucose vs 2 mol/mol glucose [41]. Increase of propionate production at low dilution rates can be related to proportionally higher requirements of carbon to maintenance energy than for assimilation of carbon to the biomass [42]. Hence, formation of propionate is favored at slow growth as more ATP is required for the maintenance. Similar ratios of acetate:propionate:butyrate 50:42:8 on AG and 84:14:2 on citrus pectin as substrates, were reported by Englyst et al. [43] in batch cultures with fecal inoculum. Thus, AG might selectively promote the propionate-producing bacteria while AP supports fermentation of sugars to acetate, which is a good substrate for butyrate producing bacteria.

Higher carbon requirement was observed also in amino acid consumptions. Most of the amino acids were completely metabolized within the whole range of dilution rates studied, except for alanine, phenylalanine, and BCAA (Figure 3). The total consumption of free amino acids increased 16–18% at 0.06 1/h compared to that at *μ* 0.2 1/h (from 0.16–0.2 up to 0.22–0.26 mol/mol, respectively) (Figure 3). Partially amino acids derived from mucin (0.2 mol-AA/mol-carb in both media) and maximal total consumption of amino acids was 0.36–0.46 mol-AA/mol-carb at *D* = 0.06 1/h. This exceeded 3.6–4.6 times the requirement for biomass synthesis under anaerobic conditions (0.1 mol-AA/mol-carb) if protein content in dry biomass is 50%, biomass yield per ATP 10 g/mol-ATP and glycolytic ATP output 2 mol-ATP/mol-glucose. The extensive use of amino acids was observed earlier in pure cultures of *B. thetaiotaomicron* [40] and can be related to shortage of energy, ammonia, or NAD^+^ regeneration. Carbon fixation through reductive TCA cycle to reduce carbon loss via CO_2_ efflux is increased with the decrease of dilution rate and can also explain the elevated requirement of NADH. Reductive or bifurcated TCA cycle has been demonstrated for several anaerobic gut bacteria such as *Clostridium butyricum* [44], *Prevotella ruminicola* [45] or *Bacteroides fragilis*, and its relatives [46]. Degradation of alanine is among the simplest ways to provide NADH or transamination of BCAA, which are common pathways present in gut bacteria according to MetaCyc database.

## Conclusions

The main advantages of the De-stat over chemostat comprise time- and cost-effectiveness in screening the impact of dilution rate on function of fecal microbiota. The De-stat study would enable to predict a change of the colon microbiota composition associated with decrease or increase of the transit rate. In-depth knowledge on gut microbiota modulation by specific growth rate is necessary to develop effective strategies for improving human health and treating microbiota-associated diseases. De-stat cultivation makes it possible to enrich and isolate the fecal microbial sub-populations adapted to fast or slow growth rates for further testing to understand the growth rate dependent community dynamics and corresponding metabolic impact. Similar approach can also be applied for other environmental parameters such as acidity or concentration of inhibitors.

Consequently, we suggest systematic analysis of the specific growth rate as an important health parameter also in nutrition studies. As the specific growth rate is related to gut transit rate, it should be taken into account when selecting dietary fibers for restoring gut bacterial balance or designing personal diets.
